# Development and validation of a prognostic model predicting symptomatic hemorrhagic transformation in acute ischemic stroke at scale in the OHDSI network

**DOI:** 10.1371/journal.pone.0226718

**Published:** 2020-01-07

**Authors:** Qiong Wang, Jenna M. Reps, Kristin Feeney Kostka, Patrick B. Ryan, Yuhui Zou, Erica A. Voss, Peter R. Rijnbeek, RuiJun Chen, Gowtham A. Rao, Henry Morgan Stewart, Andrew E. Williams, Ross D. Williams, Mui Van Zandt, Thomas Falconer, Margarita Fernandez-Chas, Rohit Vashisht, Stephen R. Pfohl, Nigam H. Shah, Suranga N. Kasthurirathne, Seng Chan You, Qing Jiang, Christian Reich, Yi Zhou

**Affiliations:** 1 Biomedical Engineering School, Sun Yat-Sen University, Guangzhou, China; 2 The Third Affiliated Hospital of Guangzhou Medical University, Guangzhou, China; 3 Observational Health Data Sciences and Informatics, New York, New York, United States of America; 4 Janssen Research and Development, Raritan, New Jersey, United States of America; 5 IQVIA, Durham, North Carolina, United States of America; 6 Department of Biomedical Informatics, Columbia University, New York, New York, United States of America; 7 Department of Neurosurgery, General Hospital of Southern Theatre Command, Guangzhou, China; 8 Department of Medical Informatics, Erasmus University Medical Center, Rotterdam, The Netherlands; 9 Department of Medicine, Weill Cornell Medical College, New York, New York, United States of America; 10 Tufts Medical Center, Institute for Clinical Research and Health Policy Studies, Boston, Massachusetts, United States of America; 11 Stanford Center for Biomedical Informatics Research, Stanford, California, United States of America; 12 Center for Biomedical Informatics, Regenstrief Institute, Indianapolis, Indiana, United States of America; 13 Department of Epidemiology, Indiana University Richard M. Fairbanks School of Public Health, Indianapolis, Indiana, United States of America; 14 Department of Biomedical informatics, Ajou University School of Medicine, Suwon, Korea; 15 Department of Biomedical Engineering, Zhongshan School of Medicine, Sun Yat-Sen University, Guangzhou, China; University of Ioannina School of Medicine, GREECE

## Abstract

**Background and purpose:**

Hemorrhagic transformation (HT) after cerebral infarction is a complex and multifactorial phenomenon in the acute stage of ischemic stroke, and often results in a poor prognosis. Thus, identifying risk factors and making an early prediction of HT in acute cerebral infarction contributes not only to the selections of therapeutic regimen but also, more importantly, to the improvement of prognosis of acute cerebral infarction. The purpose of this study was to develop and validate a model to predict a patient’s risk of HT within 30 days of initial ischemic stroke.

**Methods:**

We utilized a retrospective multicenter observational cohort study design to develop a Lasso Logistic Regression prediction model with a large, US Electronic Health Record dataset which structured to the Observational Medical Outcomes Partnership (OMOP) Common Data Model (CDM). To examine clinical transportability, the model was externally validated across 10 additional real-world healthcare datasets include EHR records for patients from America, Europe and Asia.

**Results:**

In the database the model was developed, the target population cohort contained 621,178 patients with ischemic stroke, of which 5,624 patients had HT within 30 days following initial ischemic stroke. 612 risk predictors, including the distance a patient travels in an ambulance to get to care for a HT, were identified. An area under the receiver operating characteristic curve (AUC) of 0.75 was achieved in the internal validation of the risk model. External validation was performed across 10 databases totaling 5,515,508 patients with ischemic stroke, of which 86,401 patients had HT within 30 days following initial ischemic stroke. The mean external AUC was 0.71 and ranged between 0.60–0.78.

**Conclusions:**

A HT prognostic predict model was developed with Lasso Logistic Regression based on routinely collected EMR data. This model can identify patients who have a higher risk of HT than the population average with an AUC of 0.78. It shows the OMOP CDM is an appropriate data standard for EMR secondary use in clinical multicenter research for prognostic prediction model development and validation. In the future, combining this model with clinical information systems will assist clinicians to make the right therapy decision for patients with acute ischemic stroke.

## Background and purpose

Hemorrhagic transformation (HT) is a complication of ischemic stroke that occurs after clinical therapy and is often associated with increased mortality and disability[1]. HT includes symptomatic hemorrhages that are associated with clinical worsening and those that are asymptomatic. Following ischemic stroke, the total reported spontaneous HT occurrence ranges from 3.2% (11/624) to 43.3% (61/310) and symptomatic HT ranges from 0.6% (2/624) to 7% (20/620) [2]. Electronic health records and administrative claims databases are valuable resources for re-evaluating HT incident rate base on large papulation.

Besides the incident rate need, to identify the prediction factors for HT after ischemic stroke has wide usage for clinical practices. Although HT risk factors have been previously reported, many findings are contradictory making the understanding of contributory factors of developing HT complex. For instance, the relationship between diabetes and developing HT is conflicting. In experimental transient middle cerebral artery occlusion (tMCAO) models, diabetic rats seemed to exhibit increased HT after endovascular thrombectomy than normoglycemic rats [3,4]. But other studies have come out with the opposite conclusion. Lee SH et.al. found no correlation between HT and risk factors that were significantly associated with HT, including age, sex, history of hypertension, diabetes, microbleeds, concomitant antiplatelet use, and initial infarction volume [5]. Similarly, the correlation between high lipid profiles and risk of HT is largely inconsistent with only sporadic findings to suggest an association [6–10]. A variety of other factors, including age, gender, a high National Institutes of Health Stroke Scale (NIHSS) score, fibrinogen concentration, lowered platelet count, reperfusion time, pregnancy, and cerebral microbleeds are reported to be associated with HT following acute ischemic stroke [5,11,12]. It is difficult to know which of these risk factors separate patients who have disproportionately higher HT risk from those with decreased risk of HT [13].

Clinicians face tough treatment choices to minimize the risk of early ischemic stroke recurrence. Patients are routinely prescribed anticoagulant or antiplatelet therapy after tissue plasminogen activator (tPA) treatment [14] This reduces the risk of recurrent ischemic stroke, but inversely increases the risk of developing HT [15]. A prediction model for HT after acute ischemic stroke maybe an important clinical tool to understand the impact of therapeutic regimen selections on patient’s prognosis. We propose using a data-driven approach to define the important clinical factors by incorporating all potential conditions, drugs, procedures, observations or measurements as candidate risk factors for developing HT.

At the end of 2016, the US Congress passed the "21st Century Act cure”, which approved the use of “real-world evidence” to replace traditional clinical trials for expanded indication testing[16], established the significance of real-world study (RWS) based on large number of EMR data. The purpose of this study was to use a large-scale machine learning approach to develop a model that can predict a patient’s individual risk of HT within 30 days of initial ischemic stroke for those aged 45 or older base on the real-world observational EMR data. We also investigate extensibility of this prediction model across diverse clinical settings by externally validating the model on ten different datasets.

## Methods

All method’s code and materials have been made publicly available through GitHub and can be accessed at https://github.com/OHDSI/StudyProtocols/tree/master/plpLiveValidation.

### Source of data

For both model development and model evaluation we used longitudinal observational health data from the research network called Observational Health Data Sciences and Informatics (OHDSI). In the OHDSI network, these federated datasets consist of routinely collected health data (i.e., electronic healthcare records and insurance claims records) that can be used to learn new health insights. The analysis was executed across 11 data sources from three countries and all were mapped to the Observational Medical Outcomes Partnership Common Data Model (OMOP CDM) schema [17]. The OMOP CDM provides a homogeneous format for healthcare data and standardization of underlying clinical coding systems that thus enables analysis code to be shared across participating datasets in the network [18]. Each participating site (Janssen Research & Development, IQVIA, Stanford University, and Regenstrief Institute/Indiana University) obtained institutional review board approval for the study and used de-identified data and therefore the study was determined to be exempt from human subjects research review. Informed consent was not necessary at any site. The characteristics of the datasets from each site are summarized in Table 1 below. Full descriptions of each contributing data source can be found as a table in the S1 Table. The full documentation of the OMOP CDM schema is publicly available through GitHub and can be accessed at https://github.com/OHDSI/CommonDataModel/wiki.

**Table 1:** Patient-level characteristics across data sources.

Data Source	Coverage	Data Type	No. of Patients	%		Time, year (y)	
				Female	Male	Start	End
**Optum de-identified Electronic Health Record Dataset (EHR)** ^a^	USA	Electronic Health Records	93,423,000	54.0	46.0	2006	2018
**IBM MarketScan**^®^ **Commercial Database**	USA	Claims	142,660,000	51.2	48.8	2000	2018
**IBM MarketScan**^®^ **Medicare Supplemental Database**	USA	Claims	9,964,100	55.3	44.7	2000	2018
**IBM MarketScan**^®^ **Multi-State Medicaid Database**	USA	Claims	26,299,000	56.8	43.2	2006	2017
**Japan Medical Data Center (JMDC)**	Japan	Claims	5,550,200	53.8	46.2	2005	2018
**IQVIA Disease Analyser Germany**	Germany	Outpatient Primary Care	36,078,000	56.5	43.5	1992	2018
**IQVIA Hospital Charge Data Master**	USA	Hospital Claims	88,815,000	56.1	43.9	2007	2018
**IQVIA PharMetrics Plus**	USA	Claims	153,008,000	50.9	49.1	2010	2018
**IQVIA LRxDx Open Claims**	USA	Pre-adjudicated Pharmacy and Medical Claims	654,052,000	53.0	47.0	2010	2019
**Stanford Medicine Research Data Repository (STaRR)**	USA	Electronic Health Records	3,113,080	53.9	46.1	2000	2018
**Regenstrief Institute, Indiana Network of Patient Care**	USA	Electronic Health Record	19,420,000	46.5	53.5	2005	2019

Table 1 Shows the Characteristics of the 11 datasets we studied.

^a^Dataset used to develop prediction model

### Study design

This study followed a retrospective multi-center observational cohort design[19]. We published our protocol online, it can be found at https://github.com/OHDSI/StudyProtocols/tree/master/plpLiveValidation.

#### Study population

Our target cohort consisted of patients with a first ever ischemic stroke event and who were aged 45 years or older at the time of the event. The index date is the date of the initial ischemic stroke. The patients were also required to have at least 365 days of observable time prior to the index and either an additional 30 days post index or a HT within the 30 days. Patients could not have a cerebral hemorrhage recorded within the prior 30 days or on the day index. We excluded patients who had a cerebral hemorrhage record on the same day as the ischemic stroke, since in those cases it is impossible to discern whether these are independent or sequential events. Records prior to the index date (the date of initial ischemic stroke) are used to construct candidate predictors and those after index to identify whether they experienced the HT within 30 days.

We defined ischemic stroke according to the Trial of Org 10172 in Acute Stroke Treatment (TOAST) subtype classification system [19]: (1) large-artery atherosclerosis, (2) cardio embolisms, and (3) small-vessel occlusion (lacunar infarctions). We did not include TOAST subtype (4) stroke of other determined etiology and (5) stroke of undetermined etiology due to constraints in clinical coding and validation processes on secondary data sources.

We identified these patients in the databases through the occurrence of records with OMOP standard concepts indicating these conditions. The stroke record of capture was created through the OMOP CDM by using the CONDITION_OCCURRENCE table which captures all observed conditions related to a patient as captured within the source data (e.g. insurance claims, electronic health records, hospital charge master). Observation windows were constructed using the OMOP CDM OBSERVATION_PERIOD table which is a table that contains records which uniquely define the spans of time for which a person is observed and their clinical events are being captured within the source data, even if no events in fact are recorded (e.g. healthy patient with no healthcare interactions). Detailed documentation of the OMOP CDM conventions and technical processes for mapping to the common data model is publicly available through the OHDSI Community GitHub [20,21]. A detailed list of clinical concepts (diagnosis) used to construct the target cohort definition and related exclusion criterion in this study are provided in as S2 Table.

#### Outcome measures

Our outcome is HT which we clinically defined as a symptomatic intracerebral hemorrhage occurring within one to 30 days after the initial ischemic stroke. We defined intracerebral hemorrhage as any non-chronic intracranial hemorrhage that did not originate from a trauma or aneurysm. We identified these patients in the databases through the occurrence of records with concepts indicating these conditions. A detailed list of the clinical diagnosis concepts used to construct the outcome definition and related exclusion criterion in this study are provided as S3 Table.

### Statistical analysis

Our analysis followed the standardized framework created by Reps et al [22] for patient-level prediction model development and evaluation in observational healthcare data. The framework is designed to ensure model development and evaluation are transparent and reproducible.

We developed our prediction model using Optum de-identified Electronic Health Record Dataset (Optum EHR), a large US Electronic Health Record database. We believed these data would sufficiently capture the target population and outcome as these data contained all ages and genders. This is an important consideration as insufficient representation of diversity in the training set could limit the model’s performance [22]. We chose to develop a Lasso Logistic Regression model as empirical evidence supports that this type of model generally performs wells and has greater parsimony than other machine learning models such as classification trees, random forests, artificial neural networks, and support vector machines [23]. We used a standard split of 75% of the data to train the model and the remaining 25% of the data (the hold out set) was used for internal validation. To select the optimal regularization hyper-parameter, we performed three-fold cross validation on the training dataset to search various regularization values and selected the regularization value that maximized the area under the receiver operating characteristic curve (AUC) [22]. On the 75% training data we used 3-fold cross validation to pick the hyper-parameter. Considering the data are large, using 66% of the data for each fold to train the model is sufficient to get near optimal models. To make the study more sensitivity we investigated the impact of this choice by repeating the model development on the 75% training data but using 5-fold and 10-fold cross validation when picking the optimal hyper-parameter. The discrimination was similar when using 3, 5 or 10 folds.

The overall model performance was also evaluated using the AUC. Sensitivity, specificity and positive predictive value were also calculated at different predicted risk cut-offs. The calibration was inspected via a plot showing the mean predicted risk against the fraction of patients with the outcome when the data are partitioned into deciles based on predicted risk.

In additional to internal validation, we implemented the model across 10 diverse datasets to conduct an external validation and gain insight into model transportability. The software used to develop and evaluate the model was the OHDSI Patient-Level-Prediction, an open-source patient level prediction modeling R package that programmatically expresses the patient-level prediction framework. The Patient-Level-Prediction package is externally published and maintained by the OHDSI community[24]. Using this framework, our HT risk model can be freely downloaded and consistently applied to any OMOP CDM-compliant observational database.

All our analytic code is publicly available at GitHub. The study package, which contains the specific cohort definitions used in this analysis, can be accessed at https://github.com/OHDSI/StudyProtocols/tree/master/plpLiveValidation.

### Candidate predictors

We identified a set of candidate predictor variables including demographics such as gender and age, collapsed into 5-year groupings. We also created an ordinal variable for the calendar month in which the ischemic stroke occurred, reflecting potential seasonal dependencies. In addition, we took all condition, drug, procedure, measurement, and clinical observations across the datasets and created binary variables–where a 1 represented the presence of a variable and a 0 represented the absence thereof. The absence of a patient’s condition during a valid observation period was inferred to mean that the clinical event did not occur. We defined three distinct observational time windows for binary variables which indicated the presence or absence of all conditions, drug, procedure, measurement and clinical observation: (1) in the 30 days prior to the target index (i.e. ischemic stroke), (2) in the 365 days prior to the target index or (3) any time prior to the target index. For example, patients with a record for type 2 diabetes in the prior 30 days would have a value of 1 corresponding to the variable “condition record in the prior 30 days: Type 2 diabetes” and patients without a type 2 diabetes record in the prior 30 days would have a value of 0. Variables were constructed for the three different time periods and for every condition, drug, procedure, measurement or clinical observation recorded for any patient in the target population. The hierarchy of variables is defined as part of the OMOP Standard Vocabularies [21] semantic classification system. It is standard across all OMOP CDMs and uses the same ontologies. Although this resulted in thousands of candidate predictors, Lasso Logistic Regression method adds a penalty to model complexity that generally leads to a smaller subset of candidate predictors being included and greater parsimony in the final model [25].

## Results

In total, there were 621,178 ischemic stroke patients in the development database, of which 5,624 had HT within 30 days of initial ischemic stroke. In the internal validation of the risk model, an AUC of 0.75 was achieved. The incidence of HT within 30 days of initial ischemic stroke varied between 0.1% and 1.79% across the datasets. In total, there were 5,515,508 ischemic stroke patients across 10 external validation databases, of which 80,777 had HT within 30 days of initial ischemic stroke. The discriminative performance of the models developed in the different datasets are presented in Table 2. The model and results are publicly available and can be viewed interactively from http://data.ohdsi.org/plpLive18Study/.

**Table 2:** Model performance across OHDSI data network.

Database	T (Test)	O (Test)	Incidence (%)	AUROC	AUPRC
**Optum de-identified Electronic Health Record Dataset (EHR)** ^a^	621,178 (155,259)	5,624 (1,406)	0.91	Test: 0.75 Train: 0.79	Test: 0.04 Train: 0.06
**IBM MarketScan**^®^ **Commercial Database**	274,384	4,836	1.76	0.76	0.07
**IBM MarketScan**^®^ **Medicare Supplemental Database**	441,939	6,772	1.53	0.72	0.05
**IBM MarketScan**^®^ **Multi-State Medicaid Database**	151,876	1,629	1.07	0.75	0.04
**Japan Medical Data Center (JMDC)**	20,181	31	0.15	0.7	0.05
**IQVIA Disease Analyser Germany**	41,311	45	0.11	0.5	0.02
**IQVIA Hospital Charge Data Master**	191,036	2011	1.05	0.68	0.02
**IQVIA PharMetrics Plus**	556,151	9951	1.79	0.78	0.11
**IQVIA LRxDx Open Claims**	4,331,167	54,973	1.27	0.60	0.03
**Stanford Medicine Research Data Repository (STaRR)**	7,930	142	1.79	0.76	0.12
**Regenstrief Institute, Indiana Network of Patient Care**	55,684	387	0.69	0.69	0.03

Table 2 shows the model performance across the11 datasets we studied. T = Target cohort, O = Outcome cohort, AUROC = Area under the receiver operating curve and AUPRC = Area under the precision recall curve.

^a^ Development database

**Fig 1:**
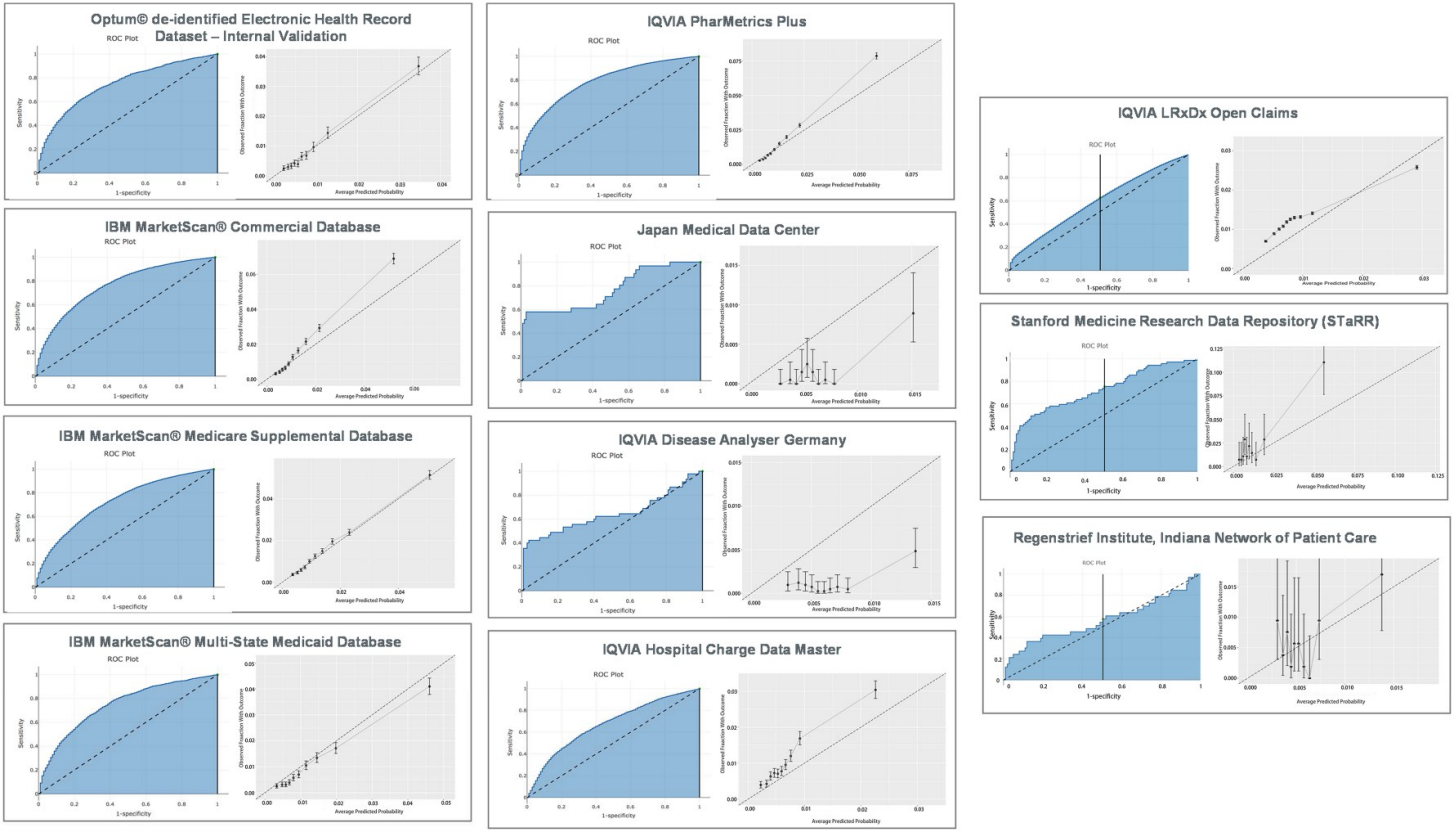
Model performance across OHDSI network sites. Fig 1 shows the receiver operating characteristic plot and calibration plot for the internal and external validation.

Out of 169,967 candidate predictors, only 612 were selected into the final model. History of other craniocerebral hemorrhage (intracranial hemorrhage, cerebral hemorrhage, spontaneous cerebral hemorrhage), cerebral edema, and compression of brain prior to developing ischemic stroke are the top three covariates contributing to the model predicting HT in a patient. Full list of the predictors also presented as a table in the S4 Table. The ROC and model calibration plots are presented in Fig 1. The model had a mean AUC of 0.71, ranging between 0.60–0.78 across network sites.

We investigated whether a simpler model could be developed by using a higher number of folds during cross validation (5 and 10) to pick the optimal regularization hyper-parameter as the model will likely pick more regularization when the folds increase. The model trained using the hyper-parameter from 5-folds cross validation still had 578 variables and 10-folds still had 557 variables. For the model to achieve a high discrimination, it seems it needs to include a large number of variables.”

This model can be used clinically to identify the 10% of patients assigned a risk of 0.0028 or lower as being low risk (risk ~0.025%) and the 6.8% of patients assigned a risk of 0.02 or greater as being high risk (risk ~4.4%). Table 3 details how we can adapt thresholds of the model to identify assigned risk.

**Table 3:** Patients with risk greater than 1% population average risk and patients with risk less than 1% population average risk.

	Predicted risk	This percentage of patients would be flagged	Risk in flagged group (%)	Risk relative to population
**If we flag patients as high risk with a predicted risk > =**	0.060	1%	10.0	10
	0.020	6.8%	4.4	4.4
	0.013	14%	3.1	3.1
	0.000	100%	1.0	1
**If we flag patients as low risk with a predicted risk <**	0.003	10%	0,025	1/40
	0.005	38%	0.12	1/8
	0.013	86%	0.47	1/2
	0.000	100%	1.0	1

Table 3 illustrates various cut off values. Flagging patients as high risk with a predicted risk higher than certain predicted risk could be used to identify a subset of the patients that have a higher than average risk. Flagging patients as low risk lower than certain predicted risk could be used to identify a subset of the patients that have a lower than average risk.

## Discussion

This study developed a predictive model for symptomatic HT, a deadly complication of ischemic stroke, based on the routinely collected observational health data. According to the prior research, the incident rate of symptomatic HT ranges from 0.6%(2/624) to 7%(20/620)[2], the study population of HT always limited to a very small one. As a result of that, it is difficult to develop a model to predict HT by a normal epidemiological investigation. In our study, due to the size of the database (total 93,423,000 patients, 621,178 patients have ischemic stroke), there were over 6,000 cases of HT within 30 days of ischemic stroke. This provided enough data to learn a model that obtained an internal AUC of 0.75. Supported by the OHDSI community and the OMOP CDM, we run an external validation across 10 datasets (total 1,232,382,380 patients; 5,515,508 ischemic stroke patients, of which 80,777 had HT) contain patients record of all races, include America, Europe, and Asia, and achieved AUC between 0.60–0.78.

The overall incidence of hemorrhagic transformation ranged between 0.11% (45/41,311)-1.79% (9951/556,151) in the 10 external validation datasets. While the incidence is lower than the prior studies report, it is still consistent with the lower range of observed incidence of symptomatic HT (0.60% to 7.0%)[2]. The incident rate was extremely low in dataset named IQVIA Disease Analyser Germany, the reason for this may be that these data focus on capturing care related to major chronic diseases (cancer, dementia, diabetes) [26] and underrepresents real hospital care of other diseases, such as hemorrhagic transformation. Other real world data sets have similar nuances. Japan Medical Data Center (JMDC) is data from 60 Society-Managed Health Insurance plans covering workers aged 18 to 65 and their dependents (children younger than 18 years old and elderly people older than 65 years old) from Japan. The main reason for its low incidence rate could be this database is designed for capturing administrative health data and cases may be underreported due to lack of specificity in billing codes used to reimburse for the condition of interest [27].

The performance varied across the 10 datasets, AUC range between 0.50 to 0.78. Databases named disease analyzer Germany with low HT prevalence may be less appropriate candidates for implementing HT risk models. The database of IQVIA LRxDx Open Claims also only had an AUC of 0.6, the reason for that could be the lack of detailed medical process records of patients. The model achieved AUC consists of higher than 0.75 in datasets Optum de-identified Electronic Health Record Dataset (EHR), IBM MarketScan^®^ Multi-State Medicaid Database, IQVIA PharMetrics Plus and Stanford Medicine Research Data Repository (STaRR) shows its transportability.

We observed several predictors that were consistent with the risk factors identified in the literature. AFib, associated with an increased risk of HT as well as worse stroke outcomes [6], was selected as a positive covariate. Similarly, prior research indicates that ‘male’ are at a greater risk of HT [1]. The model selected ‘male’ as a covariate while ‘female’ was not a selected predictor. There are other covariates selected by the model but were not reported by prior studies, for example, homonymous hemianopia as well as the use of ondansetron and dexamethasone. All other predictors we found are worth investigating in further studies because this RWE study can considered as complement of the findings from RCTs, provide valuable information on treatment practices and patient characteristics in a real-world setting [28].

The advantage of this study is that the extensive external validation has highlighted where, and where not, this model may be suitably applied. Few prediction models have been externally validated on 10 external datasets [6] and the external validation suggest our model can be transported to ‘US EHRs’ and other datasets. The respectable performance of the model in the EMR shows the possibility of inserting it into a clinical information system as a CDSS unit. The model is suitable for different datasets, for people of all races.

The model was trained with patient level characteristics, like specific demographics, medical history and prior health behaviors. As a reason for that, it goes beyond answering HT risk about average treatment effects to deliver individualized insights about a patient’s personalized future risk of experiencing HT given what the EMR data records about the patient in the past. This patient-level predictive modeling can consider as a complement to the population-level estimation of HT risk. On the other hand, because of the low incident rate of HT, it was a difficult topic to research by RCT studies. But by the real-world observational data, although the incidence of hemorrhagic transformation was only 1% in the Optum EHR data, due to the size of the database there were over 6,000 cases of HT within 30 days of ischemic stroke. This provided enough data to learn a model that obtained an internal AUC of 0.75. This indicates that the real-world observation data could help researchers solve problems that were impossible when running an RCT study.

While some data sets may contribute high counts of a condition occurrence of the outcome of interest, a high outcome count does not guarantee the data presented adequately captured the strongest candidate predictors. Therefore, model performance may be lower in data sets in which the strongest predictors are not present. This is a common challenge with model portability across real world data as we cannot always guarantee that the data the model is run on will contain the covariates that generate the most accurate prediction score. A main constraint of the current model is that it cannot be deployed without first standardizing the data for the patients at risk to the OMOP CDM. Although there is minimal information loss[25], mapping source data requires time and effort. Worthy of mention that a pilot study is working on to assist with tools transporting OMOP prediction models directly to EHRs at the bedside by FHIR (Fast Healthcare Interoperability Resources) [29]. We can expect in the further, this model can inform clinicians in the EMR system if a patient has a high risk of HT.

This study is not immune to the common challenges faced when using real world datasets. There is a potential for missing observations related to a patient who could experience the outcome event outside of the source data, such as at another health system that uses a different electronic health record than the data we studied. We acknowledge this could contribute to potential misclassification bias where a lack of observation is not congruent with a lack of event occurrence. This is a common challenge in the secondary use of data. However, this study used a limited window of follow-up which helps with minimizing the impact of this bias.

## Conclusion

A HT prognostic predict model is developed with Lasso Logistic Regression based on routinely collected EMR data can identify patients have a higher risk of HT than the population average with an AUC of 0.78. It shows the possibility to learn a risk model from observational data and OMOP CDM is an appropriate data standard for EMR second use in clinical multicenter research for prognostic prediction model development and validation. Also, we were able to show that the model can be transported to different patients across the world, despite their data being captured through different mechanisms. This study further demonstrates the application of a framework for developing transportable models that could be used to assist clinicians in identifying and managing the personalized risk for patients with a specific disease base on a patient’s EMR records. In the future, combining this model with clinical information systems will assist clinicians to make the right therapy decision for patients with acute ischemic stroke.

## Supporting information

S1 Table Full descriptions of each contributing data source. (DOCX)

S2 Table List of concepts for target cohort definition. A detailed list of clinical concepts, include patient’s diagnosis and conditions, used to construct the target cohort (patients with a first ever ischemic stroke event) definition and related exclusion criterion.
(DOCX)

S3 Table List of diagnosis concepts for outcome cohort definition. A detailed list of the clinical diagnosis concept used to construct the outcome cohort (patients have an symptomatic intracerebral hemorrhage occurring within one to 30 days after the initial ischemic stroke) definition and related exclusion criterion in this study.
(DOCX)

S4 Table Full list of selected predictors. (XLSX)
