# Supplementary material for: Development and validation of a prognostic model predicting symptomatic hemorrhagic transformation in acute ischemic stroke at scale in the OHDSI network
Source: PLoS One. 2020 Jan 7;15(1):e0226718. doi: 10.1371/journal.pone.0226718 (PMC6946584; doi:10.1371/journal.pone.0226718)
Supplement: S1 Table — (DOCX) [file pone.0226718.s001.docx]

**Supplemental Table 1. Overview of Contributing Data Sources**

| Optum© de-identified Electronic Health Record Dataset (EHR) | Optum’s de-identified Electronic Health Record data a medical records database. The medical record data includes clinical information, inclusive of prescriptions as prescribed and administered, lab results, vital signs, body measurements, diagnoses, procedures, and information derived from clinical Notes using Natural Language Processing (NLP). |
| --- | --- |
| IBM MarketScan® Commercial Database (CCAE) | Data from individuals enrolled in United States employer-sponsored insurance health plans. The data includes adjudicated health insurance claims (e.g. inpatient, outpatient, and outpatient pharmacy) as well as enrollment data from large employers and health plans who provide private healthcare coverage to employees, their spouses, and dependents. Additionally, it captures laboratory tests for a subset of the covered lives. The patients in this database are aged under 65. |
| IBM MarketScan® Multi-State Medicaid Database (MDCD) | Adjudicated US health insurance claims for Medicaid enrollees from multiple states and includes hospital discharge diagnoses, outpatient diagnoses and procedures, and outpatient pharmacy claims as well as ethnicity and Medicare eligibility. The dataset lacks lab result data. |
| IBM MarketScan® Medicare Supplemental Database (MDCR) | Represents health services of retirees (aged 65 or older) in the United States with primary or Medicare supplemental coverage through privately insured fee-for-service, point-of-service, or capitated health plans. These data include adjudicated health insurance claims (e.g. inpatient, outpatient, and outpatient pharmacy). Additionally, it captures laboratory tests for a subset of the covered lives. |
| IQVIA Hospital Charge Detail Masters (CDM) | Anonymized patient level data are sourced from hospital charge detail masters (CDM) and collected from resource management software within short-term, acute-care and non-federal hospitals. |
| IQVIA PharMetrics Plus | Patient-Centric, closed claims database of fully adjudicated pharmacy, hospital and medical claims at the anonymized patient level sourced from commercial payers. |
| Japan Medical Data Center (JDMC) | Data from 60 Society-Managed Health Insurance plans covering workers aged 18 to 65 and their dependents (children younger than 18 years old and elderly people older than 65 years old). JMDC data includes membership status of the insured people and claims data provided by insurers under contract (e.g. patient-level demographic information, inpatient and outpatient data inclusive of diagnosis and procedures, and prescriptions as dispensed claims information). |
| IQVIA Disease Analyser (DA) Germany | Data collected from physician practices and medical centers for all ages. Mostly primary care physician data however some data from specialty practices (where practices are electronically connected to each other) and some lab data is included. Key attributes include demographics, prescriptions as prescribed at brand level, diagnosis, lab measurements, actions (e.g. referrals, sick notes). |
| IQVIA LRxDx OpenClaims | Pre-adjudicated claims at the anonymized patient level collected from office-based physicians and specialists via office management software and clearinghouse switch sources for the purpose of reimbursement. We do have adjudicated claims for a subset of the medical claims data. |
| Stanford Healthcare STARR [30] | Electronic health record data derived from all patients treated as outpatients and inpatients at Stanford Hospital and Clinics from 1995 to 2019, including structured clinical data and unstructured clinical notes. |
| Regenstrief Institute, Indiana Network of Patient Care (INPC) [31] | Population-based, longitudinal, and structured coded and text data captured from hospitals, physician practices, public health departments, laboratories, radiology centers, pharmacies, pharmacy benefit managers, and payers in the Indiana Network. |
