# Supplementary material for: Development and validation of a prognostic model predicting symptomatic hemorrhagic transformation in acute ischemic stroke at scale in the OHDSI network
Source: PLoS One. 2020 Jan 7;15(1):e0226718. doi: 10.1371/journal.pone.0226718 (PMC6946584; doi:10.1371/journal.pone.0226718)
Supplement: S2 Table — A detailed list of clinical concepts, include patient’s diagnosis and conditions, used to construct the target cohort (patients with a first ever ischemic stroke event) definition and related exclusion criterion. (DOCX) [file pone.0226718.s002.docx]

**Supplemental Table 2. Ischemic Stroke Concept Set**

| **OMOP Concept Id** | **Concept Name** | **Domain** | **Vocabulary** | **Excluded** | **Descendants** |
| --- | --- | --- | --- | --- | --- |
| 443454 | Cerebral infarction | Condition | SNOMED | NO | YES |
| 761110 | Bilateral cerebral infarction due to precererbral arterial occlusion | Condition | SNOMED | NO | NO |
| 762933 | Cerebral infarction due to cerebral artery occlusion | Condition | SNOMED | NO | NO |
| 762934 | Cerebral infarction due to posterior cerebral artery occlusion | Condition | SNOMED | NO | NO |
| 762935 | Cerebral infarction due to internal carotid artery occlusion | Condition | SNOMED | NO | NO |
| 762937 | Cerebral infarction due to cerebral venous thrombosis | Condition | SNOMED | NO | NO |
| 762951 | Cerebral infarction due to anterior cerebral artery occlusion | Condition | SNOMED | NO | NO |
| 763015 | Cerebral infarction due to middle cerebral artery occlusion | Condition | SNOMED | NO | NO |
| 765515 | Cerebral infarction due to basilar artery stenosis | Condition | SNOMED | NO | NO |
| 4043731 | Infarction - precerebral | Condition | SNOMED | NO | YES |
| 4045735 | Anterior cerebral circulation infarction | Condition | SNOMED | NO | NO |
| 4045737 | Pure motor lacunar infarction | Condition | SNOMED | NO | YES |
| 4045738 | Pure sensory lacunar infarction | Condition | SNOMED | NO | NO |
| 4046237 | Infarction of optic radiation | Condition | SNOMED | NO | NO |
| 4046358 | Total anterior cerebral circulation infarction | Condition | SNOMED | NO | NO |
| 4046359 | Partial anterior cerebral circulation infarction | Condition | SNOMED | NO | NO |
| 4046360 | Lacunar infarction | Condition | SNOMED | NO | YES |
| 4046361 | Pure sensorimotor lacunar infarction | Condition | SNOMED | NO | NO |
| 4077086 | Occipital cerebral infarction | Condition | SNOMED | NO | NO |
| 4108356 | Cerebral infarction due to embolism of cerebral arteries | Condition | SNOMED | NO | YES |
| 4110189 | Cerebral infarct due to thrombosis of precerebral arteries | Condition | SNOMED | NO | YES |
| 4110190 | Cerebral infarction due to embolism of precerebral arteries | Condition | SNOMED | NO | YES |
| 4110192 | Cerebral infarction due to thrombosis of cerebral arteries | Condition | SNOMED | NO | YES |
| 4111711 | Cerebellar stroke syndrome | Condition | SNOMED | NO | NO |
| 4111714 | Cerebral infarction due to cerebral venous thrombosis, non-pyogenic | Condition | SNOMED | NO | YES |
| 4119140 | Infarction of visual cortex | Condition | SNOMED | NO | NO |
| 4131383 | Infarction of basal ganglia | Condition | SNOMED | NO | NO |
| 4138327 | Acute lacunar infarction | Condition | SNOMED | NO | NO |
| 4141405 | Left sided cerebral infarction | Condition | SNOMED | NO | NO |
| 4142739 | Thalamic infarction | Condition | SNOMED | NO | NO |
| 4145897 | Multiple lacunar infarcts | Condition | SNOMED | NO | NO |
| 4146185 | Right sided cerebral infarction | Condition | SNOMED | NO | NO |
| 4153352 | Embolic stroke | Condition | SNOMED | NO | NO |
| 4159140 | Thrombotic stroke | Condition | SNOMED | NO | YES |
| 4211509 | Cardioembolic stroke | Condition | SNOMED | NO | NO |
| 4319146 | Pituitary infarction | Condition | SNOMED | NO | NO |
| 35610084 | Cerebral infarction due to occlusion of cerebral artery | Condition | SNOMED | NO | NO |
| 35610085 | Cerebral infarction due to stenosis of cerebral artery | Condition | SNOMED | NO | NO |
| 36717605 | Silent cerebral infarct | Condition | SNOMED | NO | NO |
| 37110678 | Cerebral ischemic stroke due to occlusion of extracranial large artery | Condition | SNOMED | NO | NO |
| 37110679 | Cerebral ischemic stroke due to stenosis of extracranial large artery | Condition | SNOMED | NO | NO |
| 43530683 | Cerebral infarction due to carotid artery occlusion | Condition | SNOMED | NO | NO |
| 43531607 | Cerebral infarction due to stenosis of carotid artery | Condition | SNOMED | NO | YES |
| 44782773 | Cerebral infarction due to vertebral artery occlusion | Condition | SNOMED | NO | YES |
| 45767658 | Cerebral infarction due to thrombosis of middle cerebral artery | Condition | SNOMED | NO | YES |
| 45772786 | Cerebral infarction due to embolism of middle cerebral artery | Condition | SNOMED | NO | YES |
| 46270031 | Cerebral infarction due to occlusion of precerebral artery | Condition | SNOMED | NO | YES |
| 46270380 | Cerebral infarction due to vertebral artery stenosis | Condition | SNOMED | NO | YES |
| 46270381 | Cerebral infarction due to stenosis of precerebral artery | Condition | SNOMED | NO | YES |
| 46273649 | Cerebral infarction due to occlusion of basilar artery | Condition | SNOMED | NO | YES |

A complete view of the logic to create this Target cohort is publicly available at <http://www.ohdsi.org/web/atlas/#/cohortdefinition/1770030>.
