# Supplementary material for: Development and validation of a prognostic model predicting symptomatic hemorrhagic transformation in acute ischemic stroke at scale in the OHDSI network
Source: PLoS One. 2020 Jan 7;15(1):e0226718. doi: 10.1371/journal.pone.0226718 (PMC6946584; doi:10.1371/journal.pone.0226718)
Supplement: S3 Table — A detailed list of the clinical diagnosis concept used to construct the outcome cohort (patients have an symptomatic intracerebral hemorrhage occurring within one to 30 days after the initial ischemic stroke) definition and related exclusion criterion in this study. (DOCX) [file pone.0226718.s003.docx]

**Supplemental Table 3. Cerebral Hemorrhage Concept Set**

| **OMOP Concept Id** | **Concept Name** | **Domain** | **Vocabulary** | **Excluded** | **Descendants** |
| --- | --- | --- | --- | --- | --- |
| 376713 | Cerebral hemorrhage | Condition | SNOMED | NO | NO |
| 432923 | Subarachnoid hemorrhage | Condition | SNOMED | YES | YES |
| 439040 | Subdural hemorrhage | Condition | SNOMED | YES | YES |
| 439847 | Intracranial hemorrhage | Condition | SNOMED | NO | YES |
| 443752 | Ventricular hemorrhage | Condition | SNOMED | NO | YES |
| 4043732 | Posterior cerebral circulation hemorrhagic infarction | Condition | SNOMED | NO | YES |
| 4045743 | Massive supratentorial cerebral hemorrhage | Condition | SNOMED | NO | NO |
| 4045744 | Lobar cerebral hemorrhage | Condition | SNOMED | NO | NO |
| 4045745 | Thalamic hemorrhage | Condition | SNOMED | NO | NO |
| 4045746 | Lacunar hemorrhage | Condition | SNOMED | NO | YES |
| 4046362 | Hemorrhagic cerebral infarction | Condition | SNOMED | NO | YES |
| 4048784 | Anterior cerebral circulation hemorrhagic infarction | Condition | SNOMED | NO | YES |
| 4049659 | Subcortical hemorrhage | Condition | SNOMED | YES | YES |
| 4080892 | Intracerebellar and posterior fossa hemorrhage | Condition | SNOMED | NO | YES |
| 4108952 | Subarachnoid hemorrhage from carotid siphon and bifurcation | Condition | SNOMED | YES | YES |
| 4110185 | Intracerebral hemorrhage, intraventricular | Condition | SNOMED | NO | YES |
| 4110186 | Intracerebral hemorrhage, multiple localized | Condition | SNOMED | NO | YES |
| 4111708 | Subarachnoid hemorrhage from vertebral artery | Condition | SNOMED | YES | YES |
| 4112018 | Basal ganglia hemorrhage | Condition | SNOMED | NO | NO |
| 4129535 | Pituitary hemorrhage | Condition | SNOMED | NO | NO |
| 4144154 | Non-traumatic intracerebral ventricular hemorrhage | Condition | SNOMED | NO | YES |
| 4148906 | Spontaneous subarachnoid hemorrhage | Condition | SNOMED | YES | YES |
| 4151359 | Pars basalis hemorrhage | Condition | SNOMED | NO | NO |
| 4173481 | Cerebromeningeal hemorrhage | Condition | SNOMED | YES | YES |
| 4176892 | Cortical hemorrhage | Condition | SNOMED | NO | YES |
| 4199890 | Hematoma of brain | Condition | SNOMED | NO | YES |
| 4201094 | Cerebellar hematoma | Condition | SNOMED | NO | YES |
| 4218781 | Cerebral hemisphere hemorrhage | Condition | SNOMED | NO | YES |
| 4299377 | Intrapontine hemorrhage | Condition | SNOMED | NO | NO |
| 4319328 | Brain stem hemorrhage | Condition | SNOMED | NO | YES |
| 4326561 | Cerebellar hemorrhage | Condition | SNOMED | NO | YES |
| 37109909 | Silent micro-hemorrhage of brain | Condition | SNOMED | NO | NO |
| 40492969 | Intraparenchymal hemorrhage of brain | Condition | SNOMED | NO | YES |
| 42535423 | Spontaneous hemorrhage of deep cerebral hemisphere | Condition | SNOMED | NO | NO |
| 42535424 | Spontaneous hemorrhage of cortical intracerebral hemisphere | Condition | SNOMED | NO | NO |
| 42535425 | Spontaneous hemorrhage of cerebral hemisphere | Condition | SNOMED | NO | NO |
| 42538062 | Spontaneous intracranial hemorrhage | Condition | SNOMED | NO | YES |
| 42709921 | Hemorrhage into subdural space of neuraxis | Condition | SNOMED | YES | YES |
| 42872434 | Intracranial hematoma | Condition | SNOMED | NO | YES |
| 42873042 | Subpial intracranial hemorrhage | Condition | SNOMED | NO | YES |
| 42873044 | Hemorrhage in globus pallidus | Condition | SNOMED | NO | NO |
| 42873045 | Hemorrhage in caudate nucleus | Condition | SNOMED | NO | NO |
| 42873046 | Hemorrhage in putamen | Condition | SNOMED | NO | NO |
| 42873109 | Hemorrhage into subarachnoid space of neuraxis | Condition | SNOMED | YES | YES |
| 42873123 | Hemorrhage of intracranial meningeal space | Condition | SNOMED | YES | YES |
| 42873124 | Hemorrhage into meningeal space of neuraxis | Condition | SNOMED | YES | YES |
| 43530674 | Spontaneous cerebellar hemorrhage | Condition | SNOMED | NO | YES |
| 43530727 | Spontaneous cerebral hemorrhage | Condition | SNOMED | NO | YES |
| 43530851 | Chronic non-traumatic intracranial subdural hemorrhage | Condition | SNOMED | YES | YES |
| 44782730 | Nontraumatic intraparenchymal cerebral hemorrhage | Condition | SNOMED | NO | YES |
| 45766068 | Deep hemispheric cerebral hemorrhage | Condition | SNOMED | NO | YES |
| 45766118 | Non-aneurysmal subarachnoid intracranial hemorrhage | Condition | SNOMED | YES | YES |
| 45766119 | Perimesencephalic subarachnoid hemorrhage | Condition | SNOMED | YES | YES |
| 45766120 | Convexal subarachnoid hemorrhage | Condition | SNOMED | YES | YES |
| 45766830 | Non-aneurysmal perimesencephalic subarachnoid hemorrhage | Condition | SNOMED | YES | YES |
| 46270111 | Nontraumatic subarachnoid hemorrhage with brain compression | Condition | SNOMED | YES | YES |
| 46273491 | Spontaneous cerebral hemorrhage with compression of brain | Condition | SNOMED | NO | YES |

A complete view of the logic to create this Outcome cohort is publicly available at <http://www.ohdsi.org/web/atlas/#/cohortdefinition/1770029>.
